# Digital technology-enhanced reconstruction and rehabilitation following mandible fibular flap grafting with implants: a case report

**DOI:** 10.3389/fdmed.2025.1554315

**Published:** 2025-03-05

**Authors:** Shuang Li, Jian Liu, Jian Li, Baoheng Yin, Yuanyong Feng, Yanshan Liu, Na Bai

**Affiliations:** ^1^ The Affiliated Hospital of Qingdao University, Qingdao, China; ^2^ Department of Prosthodontics, The Affiliated Hospital of Qingdao University, Qingdao, Shandong, China; ^3^ Suqian Stomatological Hospital, Suqian, China; ^4^ Department of Oral Implantology, The Affiliated Hospital of Qingdao University, Qingdao, China; ^5^ School of Stomatology, Qingdao University, Qingdao, Shandong, China; ^6^ Department of Oral and Maxillofacial Surgery, Affiliated Hospital of Qingdao University, Qingdao, Shandong, China

**Keywords:** digital dentistry, digital facebow, dental implants, mandibular reconstruction, free fibular flap

## Abstract

**Objective:**

In this paper, we report a case of missing posterior teeth after mandibular fibular flap grafting and implant restoration by means of a digital restorative process, in the hope that the application of digital technology will help the patient to comfortably establish an adapted occlusal relationship.

**Case report:**

A 29-year-old male patient developed a left mandibular ameloblastoma measuring 35 mm × 35 mm × 25 mm, which did not invade the surrounding bone tissue, submandibular gland tissue, or lymph nodes. After resection of an 85 mm mandibular segment, the patient was reconstructed using a gastrocnemius flap transfer. He was then referred to our institution for postoperative dental implant restoration. The restorative process incorporated various digital technologies including a digital facebow, intraoral scanner, extraoral scanner, facial scanner, and CAD/CAM systems. Following 1 year post-implantation, the patient received second-stage implantation alongside autologous dermal allograft (ADM) transplantation; subsequently, a temporary prosthesis was fabricated while employing an electronic articulator to accurately transfer occlusal relationships before finalizing with permanent restorations. The integration of digital technology throughout this restorative process enhanced both precision and comfort.

**Conclusions:**

This case study offers an innovative and efficient clinical approach for addressing dentition defect following mandibular reconstruction via advanced digital methodologies.

## Introduction

Defects in the jawbone are often the result of trauma, cancer, and other reasons. Reconstruction using vascularized free flaps and segmental excision of the jaw are common techniques for fixing these bone abnormalities. Multiple teeth are frequently lost following the excision of a sizable piece or even the entire jawbone, upsetting the preexisting interactions between the soft and hard tissues. Restoring the correct occlusal connections in the jawbone that has been rebuilt is essential to functional recovery. At the moment, fibula grafting along with implant restoration has produced a number of positive clinical results (1–3). The stability of the implants is increased by the fibula's distinct double-cortical structure and bone quality ratio, which closely mimic that of the jaw. Additionally, a segmental blood supply to the free fibula flap is advantageous (4, 5), which helps to explain the high graft success rate and low rates of bone resorption (6). With the advantages of adequate bone length, the possibility of using skin islands, and the low morbidity in the donor area, the free fibular muscle flap (FFF) has become a popular and highly successful surgical procedure for reconstruction of mandibular anomalies (7). Throughout the entire course of treatment, oral prosthesis are crucial. The patient's future life and health will be negatively impacted if oral function cannot be restored without full restorative therapy. Fixed implant restoration offers major benefits over removable dentures in terms of maximizing occlusal function and restoring the structural integrity of the dental arch (8).

Creating personalized imprint trays, fixing splints with light-curing materials, and gypsum casting are some of the procedures involved in the classic implant restoration process. There is a chance that the castings could be harmed during this intricate procedure. But advances in digital technology have made every step of implant restoration easier, including the printing of three-dimensional models, computer-aided design and manufacture (CAD/CAM), and the collecting of three-dimensional data in dentistry. Different data acquisition technologies allow clinicians to obtain precise data on the soft and hard tissues of the oral and maxillofacial regions during the restoration process. These technologies include electronic facebow technology, cone-beam computed tomography (CBCT), intraoral 3D scanning, facial 3D scanning, and ICam4D photogrammetry. With the use of these technologies, suitable occlusal connections can be established by creating virtual patients, capturing mandibular movement trajectories, and accurately transferring the articulator. This method improves patient comfort during the course of therapy while cutting down on chairside operating time without sacrificing precision. Moreover, customized occlusal corrections and restoration design and fabrication are made possible by CAD/CAM technology, guaranteeing the best possible results for the completed restoration. The electronic facial arch includes intraoral scanning of the complete dental arch, recording of occlusal relationships, and the use of recorded jaw movement trajectories to alter the dynamic occlusion, effectively delivering the jaw connection in a simple, accurate and fast manner (9). Virtual jaw frames, including e-facebows, have been widely used in clinically complex cases including bruxism and TMJ to aid in the diagnostic and treatment planning phases of the case with a fully digital workflow (9, 10). Digital technology can simplify treatment planning and aesthetic design and enhance medical communication (11). However, digital 3D scanning technology does not allow for the acquisition of mucosal morphology under compression and therefore cannot be applied to implant overdentures, limiting the choice of restorative modalities. Digital techniques such as intraoral scanning, extraoral scanning, and e-arch have facilitated the restorative process by allowing patients to design appropriate restorations, but they still require multiple visits and are complex and expensive. There are still many challenges to the spread of digital technology.

This article describes a case of fibula grafting followed by an implant-fixed restoration using a variety of digital technologies. A virtual patient was created using intraoral 3D scanning, facial 3D scanning, and ICam4D photogrammetry. Accurate transfer of mandibular movement trajectories was made possible using an electronic facebow. The final restoration was successfully finished to the patient's satisfaction through personalized digital design. For patients undergoing bone restoration, this procedure provides a comfortable and effective means of restoring occlusal connections and aesthetics.

## Clinical report

**Figure 1:**
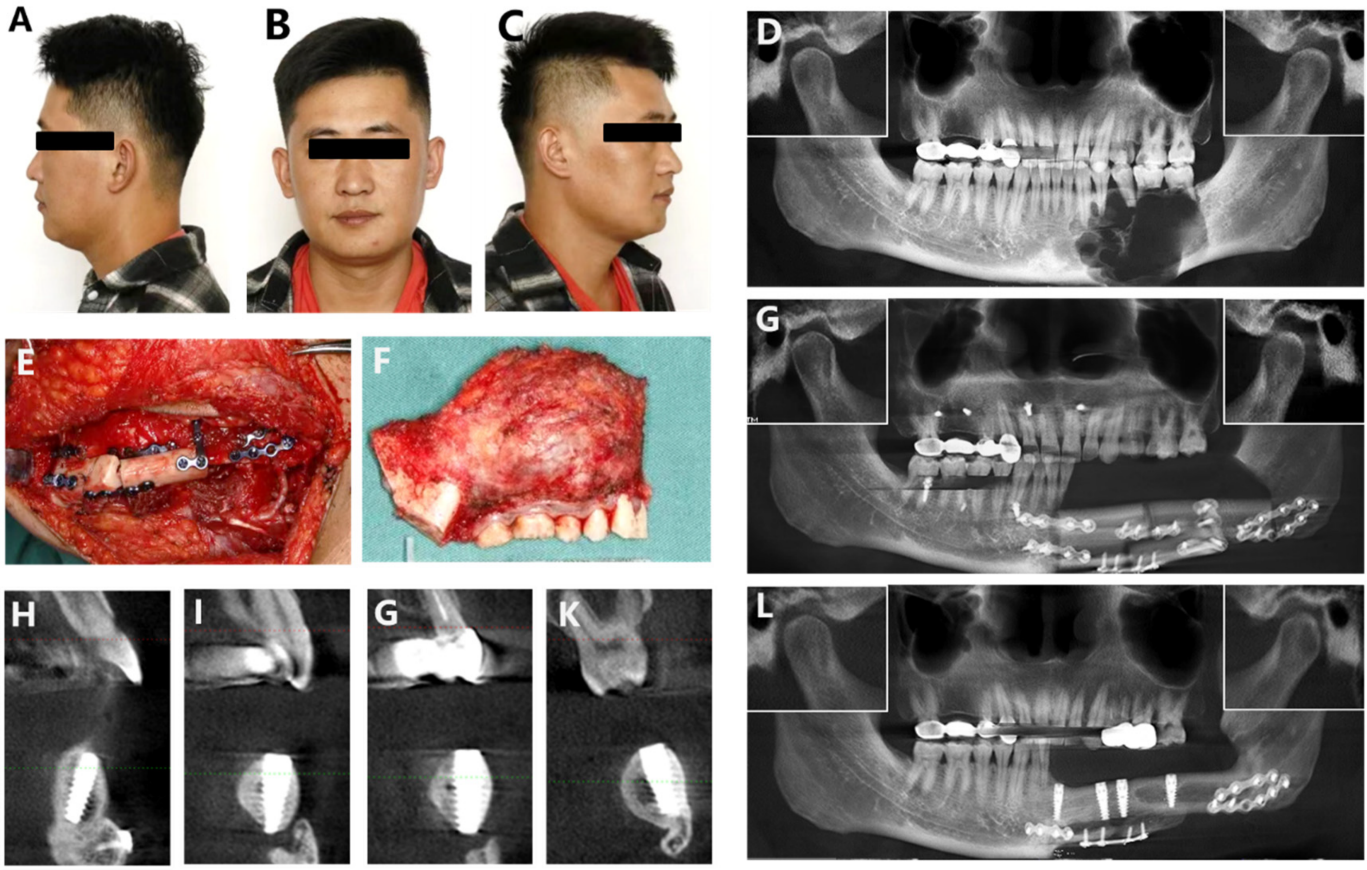
**(A–C)** Facial appearance before treatment, including front and side views. **(D)** Ameloblastoma preoperative CBCT. **(E)** Peroneal muscle flap transfer repair intraoperative. **(F)** Resection of the diseased mandibular segment. **(G)** Ameloblastoma postoperative CBCT. **(H–K)** Implant buccal and lingual imaging. **(L)** CBCT after implantation.

The patient is a 34-year-old male who needs his implant in the left mandibular fibula graft site restored. The occlusal relationship was good during the normal occlusal state before and after the bone reconstruction surgery; the position of the condyle on the normal side did not change significantly and was still located in the articular fossa, and the position of the condyle on the affected side was also located in the articular fossa, which represents that the position of the mandible did not change significantly during the surgical three-dimensional reconstruction, and the reset basically reached the preoperative position. The implant has been planted for more than a year. Three years ago, he underwent a left mandibular tumor resection, involving the excision of an ameloblastoma, at our hospital's oral and maxillofacial surgery department. Wide excision of the left mandible, segmental resection of the jaw, vascular anastomosis, transfer of the fibula myocutaneous flap for reconstruction, titanium plate and screw implantation, and mandible reconstruction were among the treatments carried out (Figure 1A–F). A year ago, he had his internal fixation device removed. Four Nobel Active implants—two 4.3 mm × 13 mm, one 3.5 mm × 13 mm, and one 4.3 mm × 11.5 mm—were placed in the left mandibular fibula graft area during that procedure (Figure 1H–K). After determining that the patient's postoperative recovery was good, the oral and maxillofacial surgeon recommended the patient to our department for restoration.

The oral and maxillofacial examination indicates a notable asymmetry in the patient's jaw and facial structure, with the left side exhibiting hypertrophy compared to the right. The left aspect of the mandible appears recessed, while the chin is deviated towards the right. The maximum interincisal opening measures two fingerbreadths and is also skewed to the right. Notably, there is an absence of tenderness or clicking sounds in both temporomandibular joint regions. The gingiva and mucosa of the left mandibular area have scar tissue. The occlusal connection is oriented to the left, and the midline is unaltered, displaying a Class I deep overbite with normal covering. The distal bone segment is slanted buccally, and teeth 31–37 are missing along with insufficient bone height in the fibula graft. The graft area's mucosa is thin and devoid of normal muscle attachment and keratinized gingiva. The gingival distance between teeth 31–36 is roughly 10 mm, while tooth 37 has insufficient occlusal space, with a gingival distance of roughly 7 mm. Teeth 14 and 16 have crowns on them, but teeth 15 and 25 are missing. A bridge restoration spans teeth 23–27. Tooth hygiene is not up to par. Clear fluid is released from the bilateral parotid duct apertures, and neither redness nor swelling is visible there. The tongue exhibits no discernible deviation following extension, remains centered, and glides freely. There is numbness on the skin of the lower left lip. There is no congestion in the pharynx, and there is no enlargement of the tonsils.

CBCT (Figure 1G) displays a left mandibular deformity that is treated with a titanium plate, screws, and a double-layer fibula graft. With the repaired bone section, the implant is well-integrated and does not exhibit any notable low-density areas. While implants 34, 35, and 37 are roughly parallel in the mesiodistal direction, implant 31's apex is inclined distally. Furthermore, implant 37 is positioned in a buccal manner.

The clinical diagnosis for this patient includes: dentition defect, jawbone deficiency, and post-fibula graft. Before initiating the treatment process for this case, we anticipate the potential challenges we may face as follows: Following fibula bone transplantation, alterations in soft and hard tissue relationships within the oral and maxillofacial region complicate impression-taking due to a complex intraoral environment. Furthermore, insufficient keratinized gingiva surrounding the implant heightens risks associated with bone resorption and peri-implantitis (12). Given abnormal mandibular movement coupled with a lack of normal occlusal relationship, accurately determining an appropriate occlusion position proves challenging. Restoration efforts are further complicated by 37 implants being positioned at an angle toward the buccal side alongside inadequate occlusal space. Additionally, traditional methods for impression-taking and articulator transfer are time-consuming, adversely affecting patient comfort.

Following resolution of the aforementioned concerns, the patient's request for a restoration that strikes a balance between practicability and aesthetics must be discussed. Preoperative impressions are obtained, and then a CBCT scan (New Tom 5G, QR, Verona, Italy) is performed in order to improve surgical precision and create a surgical plan for the second step of implantation. In implant restoration procedures, adequate width of keratinized gingiva plays a crucial role in mitigating plaque accumulation as well as minimizing local alveolar bone resorption; thus reducing peri-implantitis incidence rates. In light of deficient normal keratinized gingiva within the surgical site, it was proposed that simultaneous II-stage implantation be conducted alongside autologous dermal allograft (ADM) transplantation—secured to adjacent gingival tissues—to enhance keratinized mucosa volume at this site while expediting treatment duration. A combination of three-dimensional oral scanning technology, three-dimensional facial scanning technology, and ICam4D photogrammetry techniques were employed for precise impression acquisition; subsequently fabricating a temporary implant bridge utilizing implants 31–37. Considering tooth 27's designation as a functional cusp led to posterior teeth being arranged in reverse occlusion. The design of the second edition of the temporary restoration is influenced by the mandibular movement trajectory measured by electronic facebow measurements obtained after the temporary bridge is fitted. In order to determine the ideal occlusal connection, the jaw position and occlusal relationship are assessed for appropriateness, and any required modifications are made to the temporary restoration. The final restoration is made and fitted based on the occlusal relationship established by the temporary repair. Following placement, intraoral scans, facial scans, and electronic facebow are used to evaluate the final restoration in order to determine its overall success.

The treatment process for the patient is divided into three stages: the surgical procedure, the fabrication and trial of a temporary prosthesis, and the creation of the final prosthesis.

**Figure 2:**
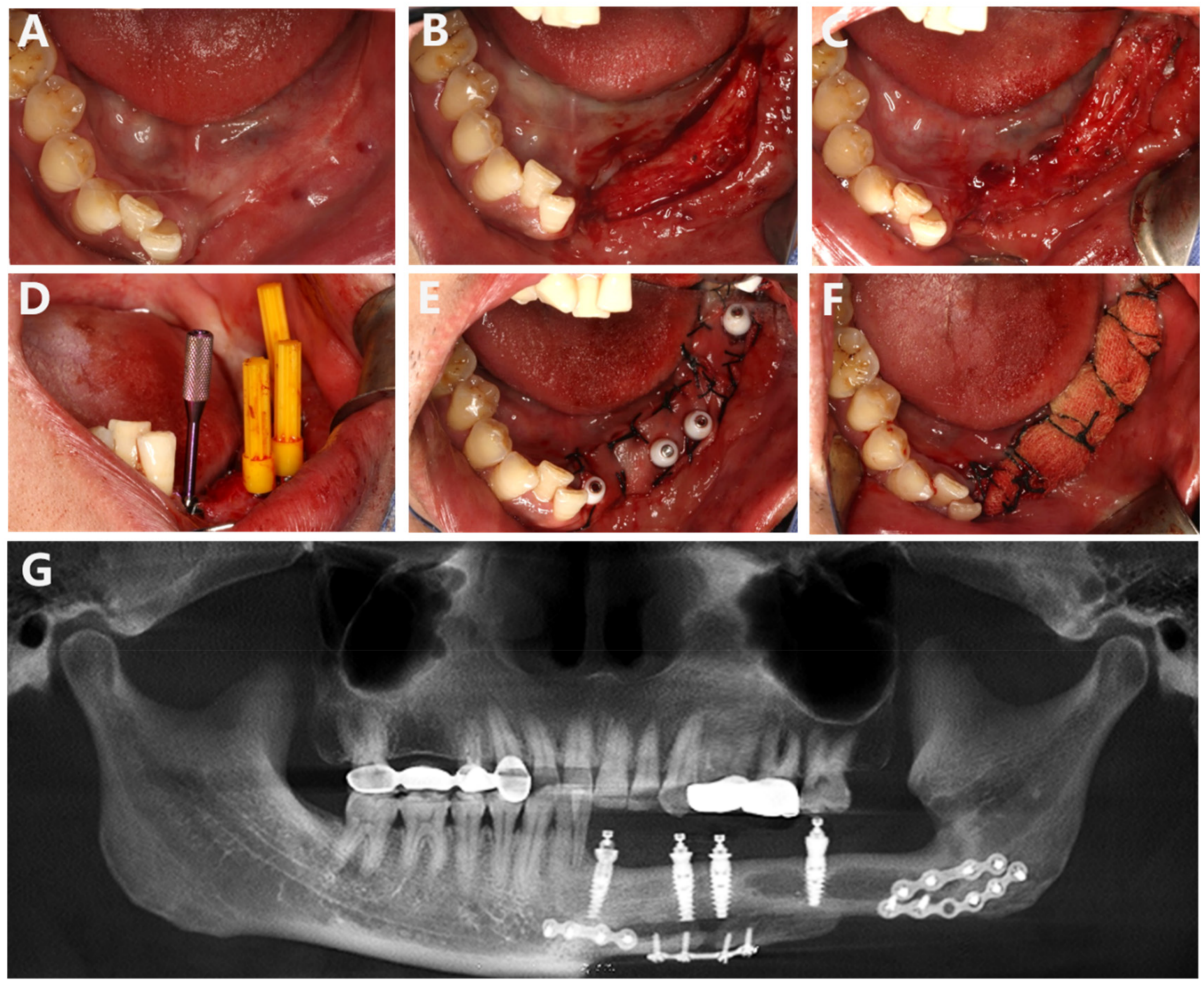
**(A)** Preoperative mucosa at the second stage of implantation. **(B)** Incision of the gingiva. **(C)** Fixation of local mucosa to the buccal and lingual periosteum. **(D)** Placement of the Multi-unit abutment. **(E)** ADM implantation. **(F)** Local pressure stabilization with iodoform gauze strips. **(G)** CBCT imaging after placing the Multi-unit abutment indicates that the abutment is properly positioned.

Based on these clinical considerations regarding patient condition, simultaneous II-stage implantation along with ADM transplantation was executed successfully alongside placement of Multi-unit abutments. A straight incision was made along the alveolar ridge crest corresponding to teeth 31–37, and the mucoperiosteal flap was carefully elevated along this incision. The physician utilized a slow-speed bur to remove bone, thereby exposing four cover screws. Subsequently, 4-0 absorbable sutures were employed to secure the mucosa to the periosteum. The cover screws were then removed. For implant position 31, a narrow-diameter multi-unit abutment with a gingival penetration height of 3.5 mm at an angle of 17° was installed; for position 34, a multi-unit abutment with a diameter corresponding to a gingival penetration height of 2.5 mm was used; for position 35, another multi-unit abutment with a diameter allowing for a gingival penetration height of 3.5 mm was placed; and finally, for position 37, a multi-unit abutment with marked diameter and gingival height of 4.5 mm was installed accordingly. The physician trimmed an allogeneic dermis (ADM) measuring approximately 3 cm × 1 cm × 2 cm and positioned it over the healing abutment site before securing it in place using protective caps on the abutments and fixing the ADM with non-resorbable silk thread (4-0). Iodoform gauze strips were subsequently secured under pressure using larger silk suture material (2-0) (Figure 2A–F). Postoperative CBCT imaging confirmed proper positioning (Figure 2G).

**Figure 3:**
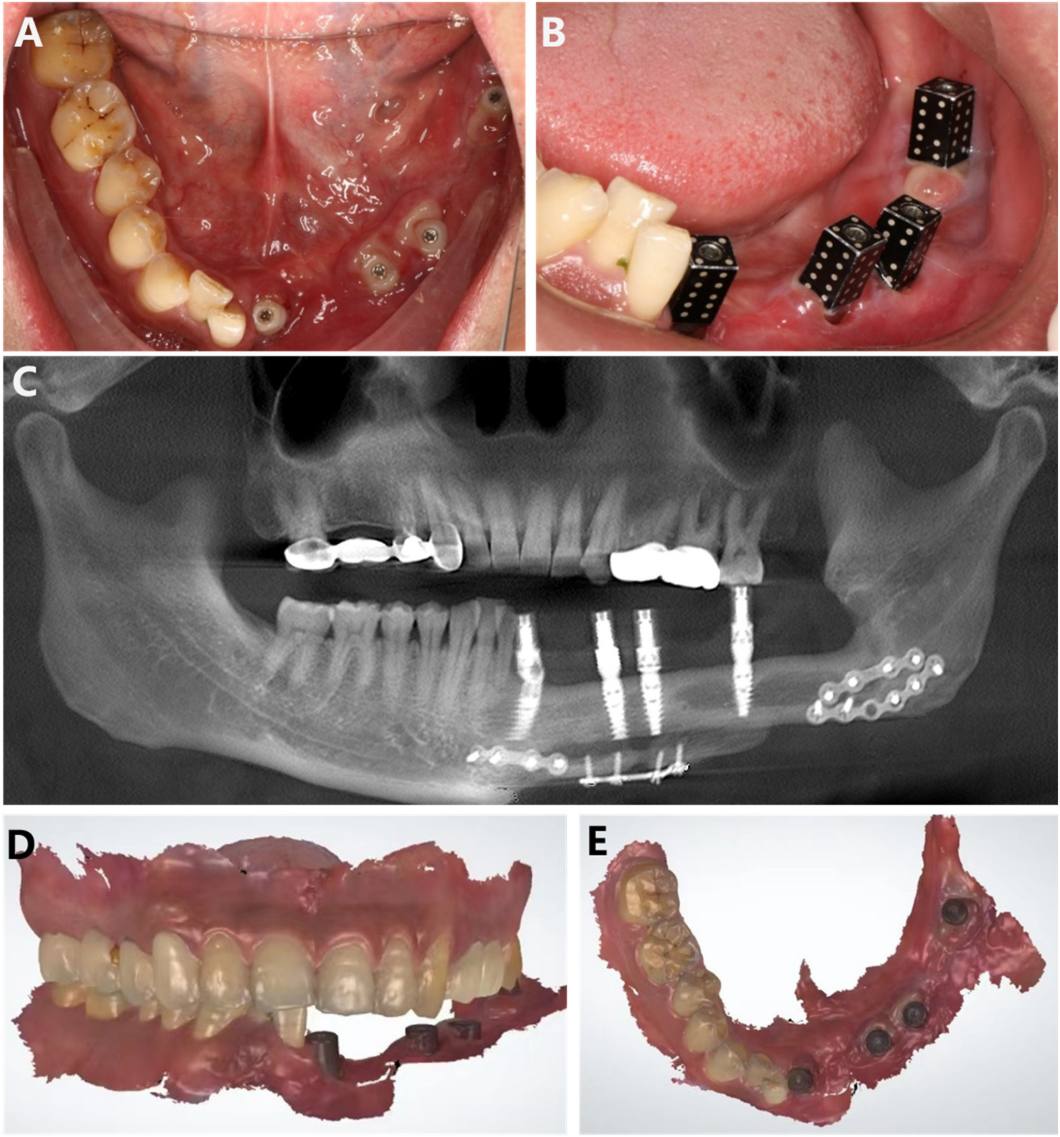
**(A)** Intraoral photo 2 months post-gum grafting, showing good healing of the ADM. **(B)** Placement of ICamBodies for ICamPosition. **(C)** CBCT imaging taken after placing the ICam4D extraoral scanning accessory. **(D,E)** Intraoral scan data.

**Figure 4:**
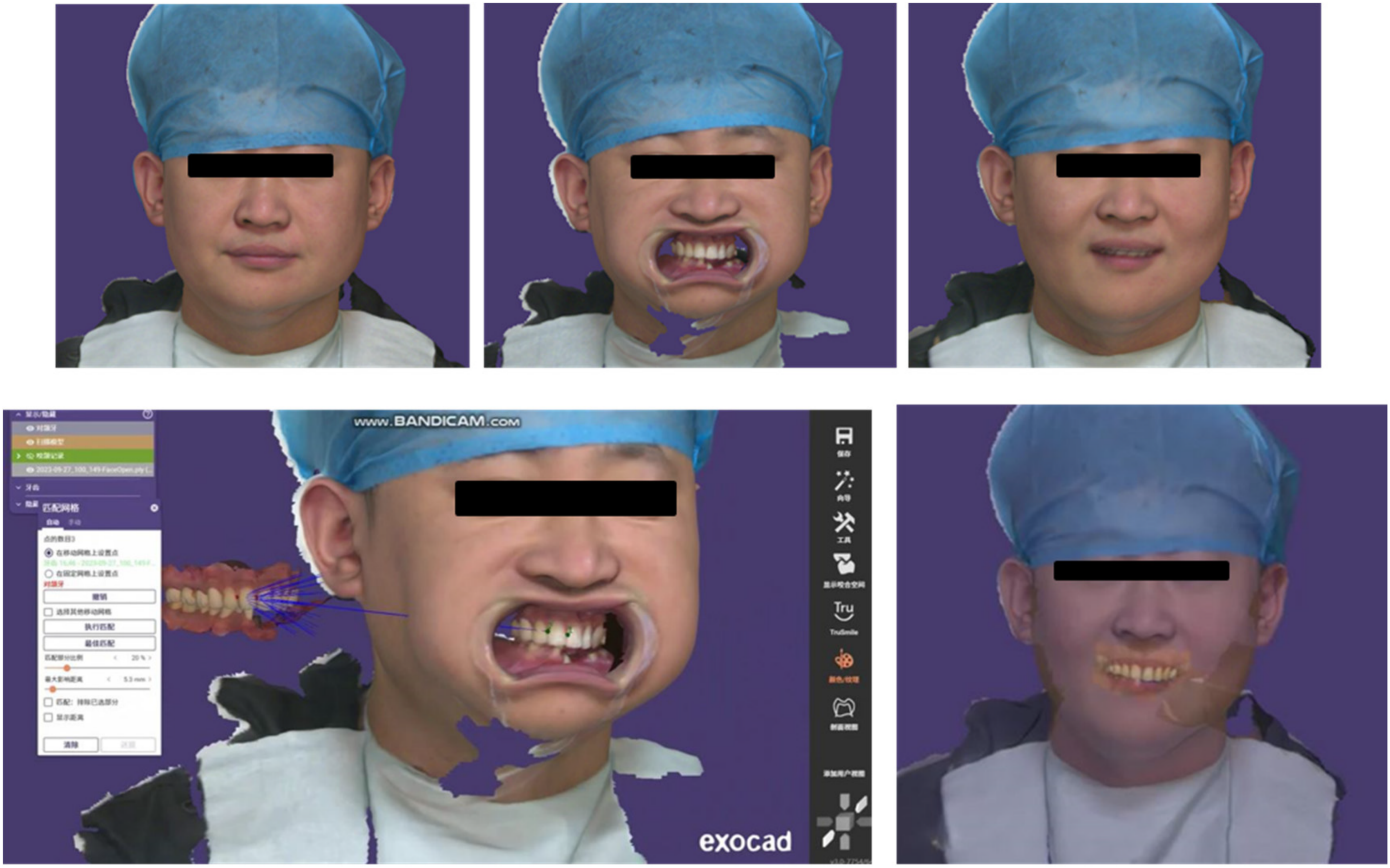
The integration of facial scan data with intraoral scan data is utilized for the digital design of the temporary restoration.

**Figure 5:**
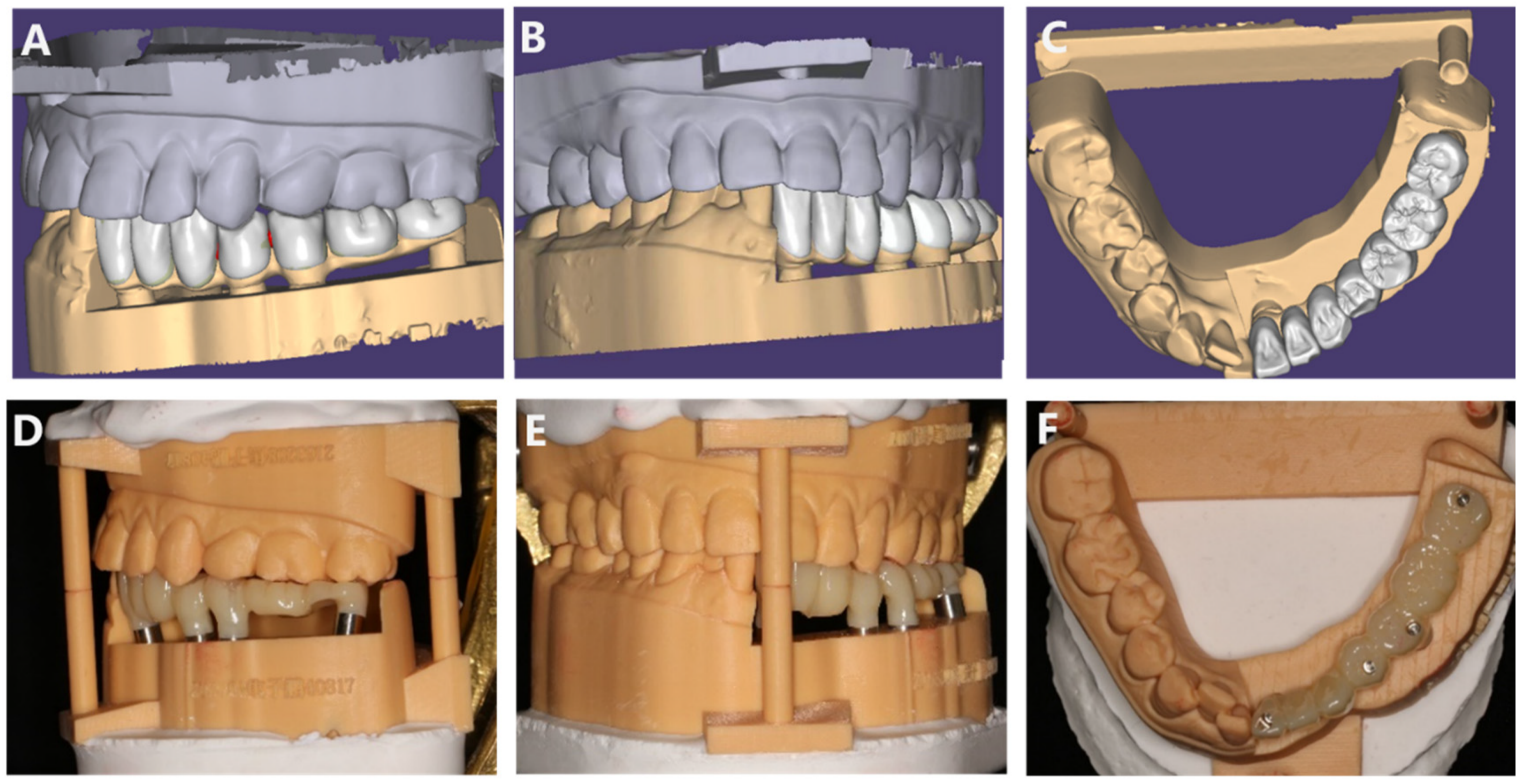
**(A–C)** Digital design of the temporary prosthesis; **(D–F)** temporary prosthesis.

During the fabrication of the temporary prosthesis, we employed a combination of ICam4D extraoral scanning, intraoral 3D scanning, and facial 3D scanning technologies to obtain accurate impressions. The ICam4D extraoral scanning technology, intraoral three-dimensional scanning, and facial t scanning techniques are jointly applied for the oral and facial impression-taking of the patient (Figure 3). Special scanning bodies, ICamBodies, are installed on teeth 31, 34, 35, and 37. The scanner is calibrated and moved slowly from one side of the patient to the other to capture the laser marking points on the scanning bodies, obtaining the relative position data of the scanning bodies and thereby determining the precise position of the multi-unit abutments. The intraoral scanning rods, ICamRefs, are replaced on teeth 31, 34, 35, and 37. The TRIOS 3rd-generation intraoral scanner (3Shape, Denmark) is employed to acquire the data of the intraoral mucosa and implant positions, which are then imported into the CAD design software (3Shape DentalSystem, Denmark). Facial features are collected using the DS FSCAN + facial 3D scanner (Shining, China), and the digital data are imported into the professional design software Exocad (version 1.6.4, Germany) to generate a three-dimensional model of the virtual patient and jointly design the temporary restoration in combination with the intraoral scanning data (Figures 4, 5).

**Figure 6:**
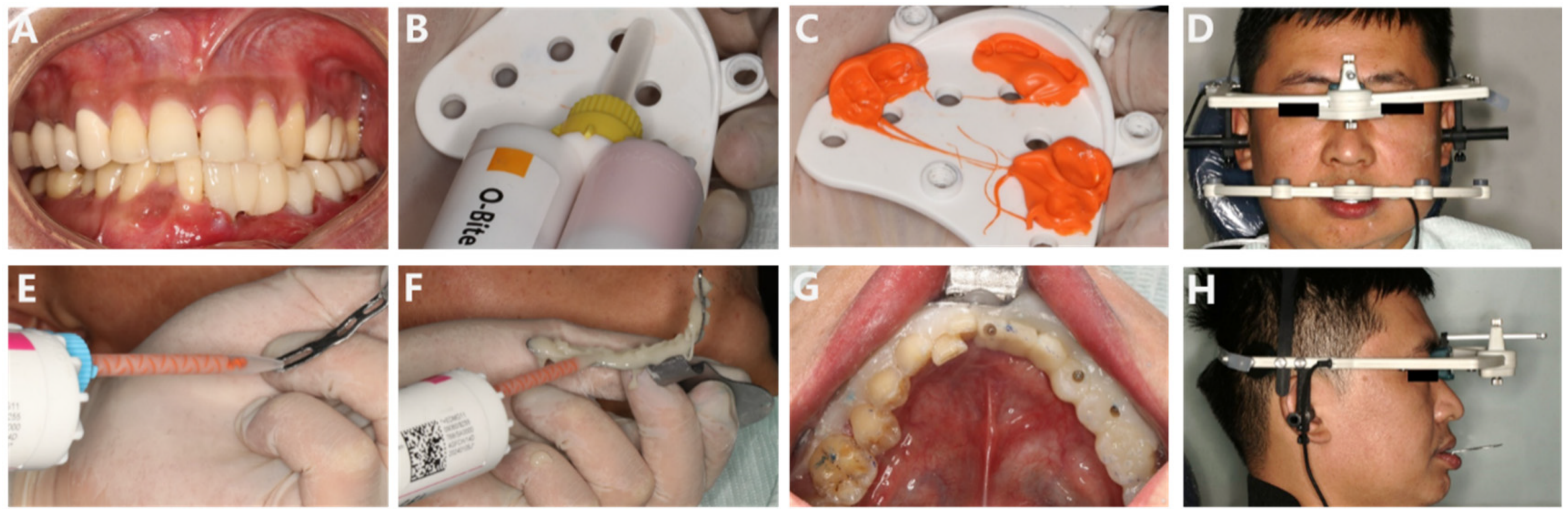
**(A–H)** Try-in of the temporary prosthesis; measurement and transfer of the mandibular movement trajectory using the electronic facebow.

**Figure 7:**
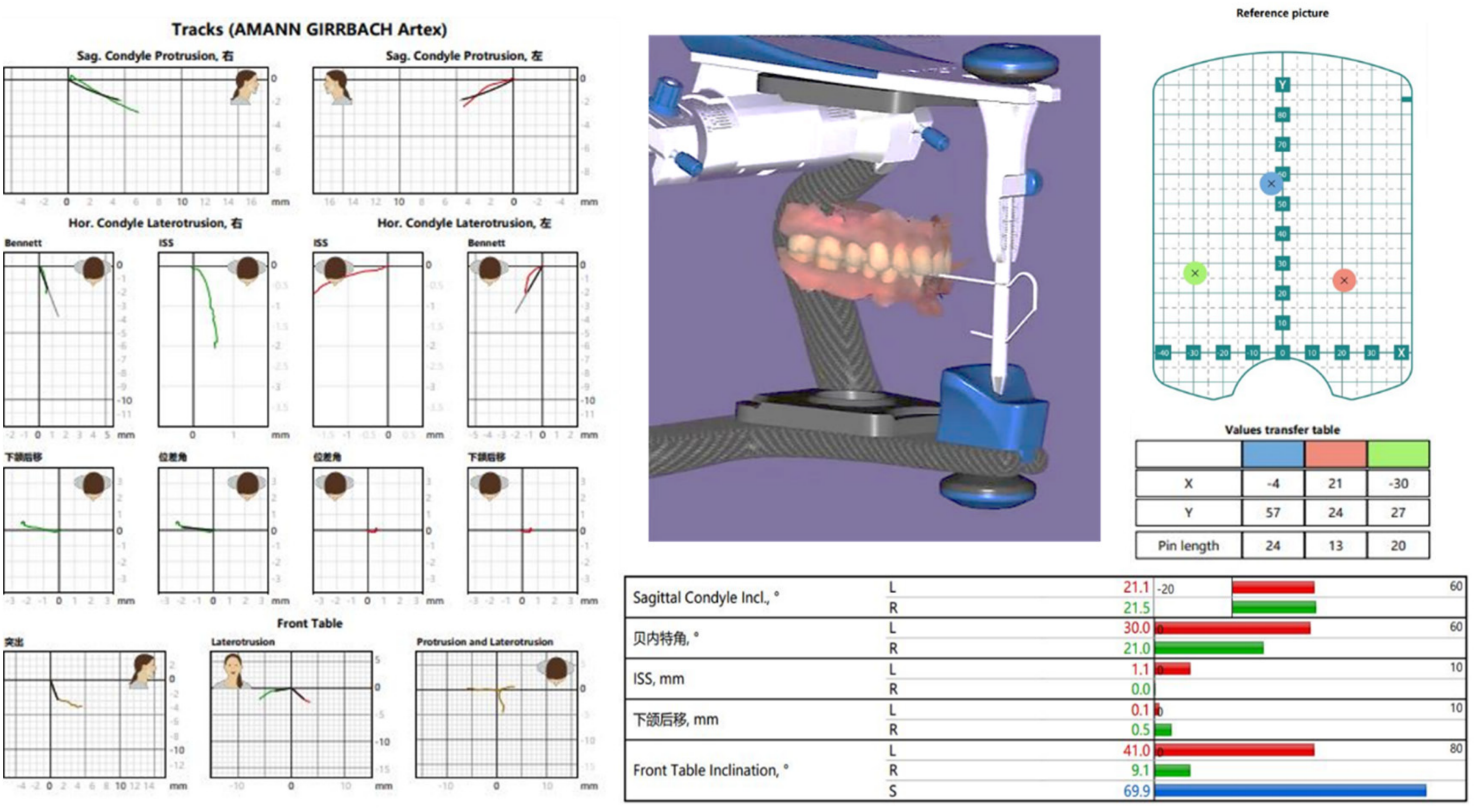
Analysis of digital facebow data, including functional mandibular movement trajectories and relevant averages.

The patient is instructed to try on the temporary restoration and perform centric, lateral, and protrusive occlusion. Due to bone reconstruction, occlusal discrepancies may occur, which are then adjusted appropriately to ensure consistent height in the bilateral mandibular posterior teeth. Once the position is confirmed, the trajectory of the mandibular movements is measured and transferred using the JMA Optic electronic facebow (Zebris JMAnalyser+, Germany) (Figures 6, 7). In the clinical examination, the patient had good lateral and anterior mandibular movements with high repeatability and stability of movements. According to the analysis of the mandibular movement facial arch trajectory chart, the bilateral movements were not completely symmetrical. When the temporary restoration was put in, the mandibular movement trajectory suggested that the patient's incisal guidance angle was larger and the overlay was deeper. When the final restoration was placed, the above situation was relieved, and the movement trajectory was smoother.

Based on the information provided, a 31–37 implant bridge restoration is proposed. Taking into account the morphology and movement factors of the left mandible, the implant denture should be designed as a posterior tooth reversal. The positioning of the 37 implant is suboptimal, resulting in insufficient occlusal space, and the occlusal surface will be designed with zirconium.

**Figure 8:**
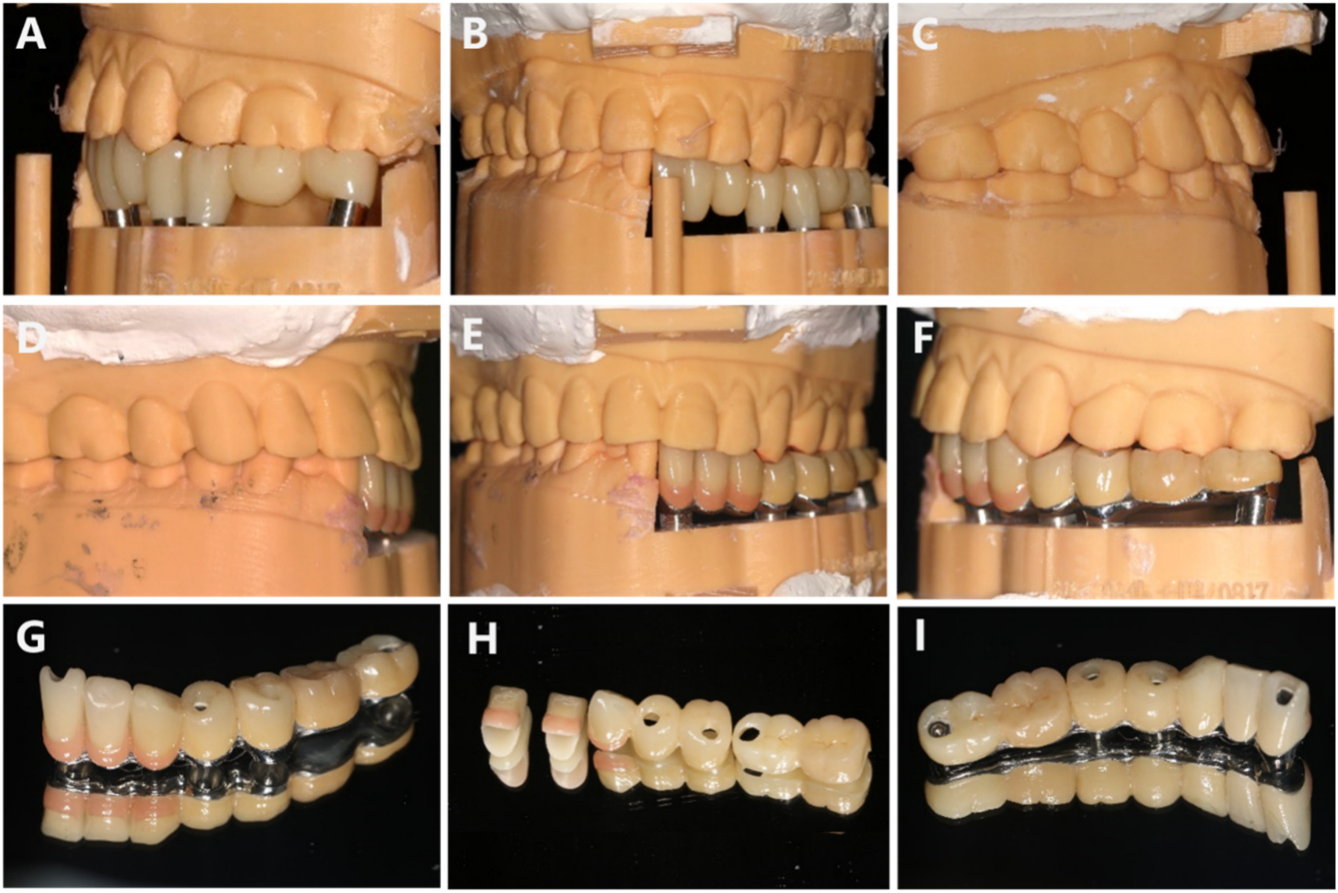
**(A–C)** Second edition temporary restoration. **(D–I)** Final restoration.

To provide a more precise and personalized restoration for the patient, we developed a second version of the temporary prosthesis based on the measurements from the first version for trial fitting. During the try-in of the second temporary restoration, the seating went smoothly. The final restoration will be fabricated as a split crown bridge after returning to the lab, incorporating gingival porcelain. Shade matching for both the crowns and gingiva will be performed to create the final restoration (Figure 8A–C).

**Figure 9:**
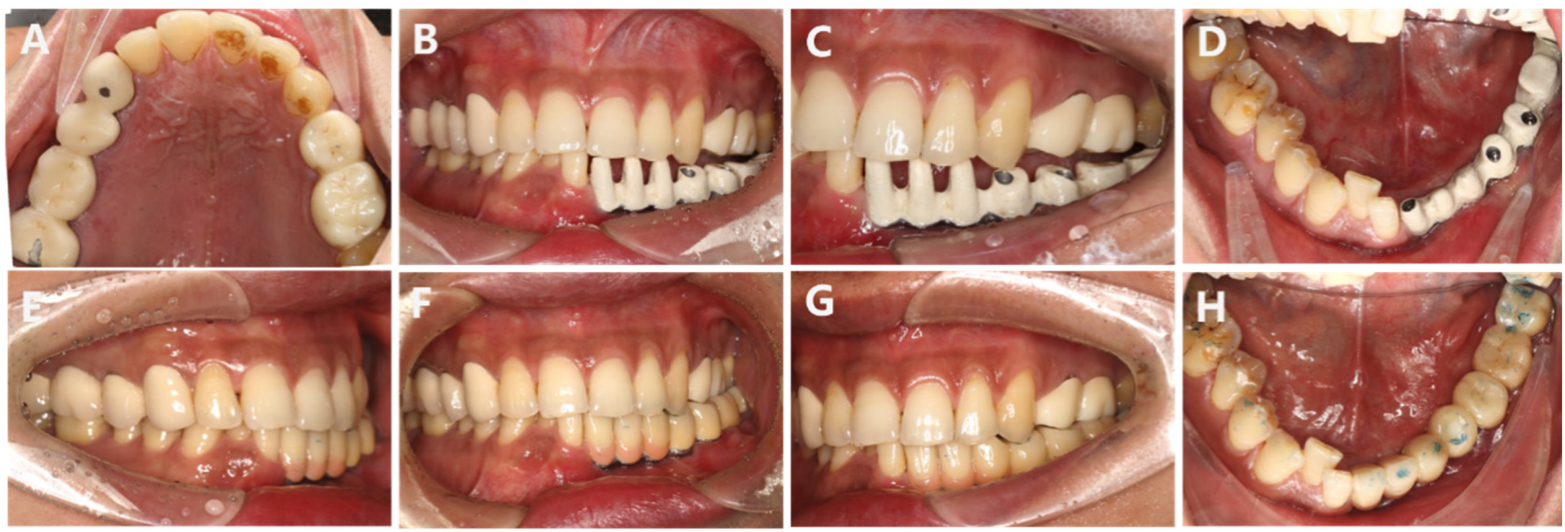
**(A–D)** Intraoral photograph of the monolithic bridge try-in; the bridge is passively seated. **(E–H)** Intraoral try-in of the final restoration. The occlusal relationship is satisfactory, and the coverage is adequate. The patient expresses satisfaction with the aesthetic outcome, and the restoration is now complete.

**Figure 10:**
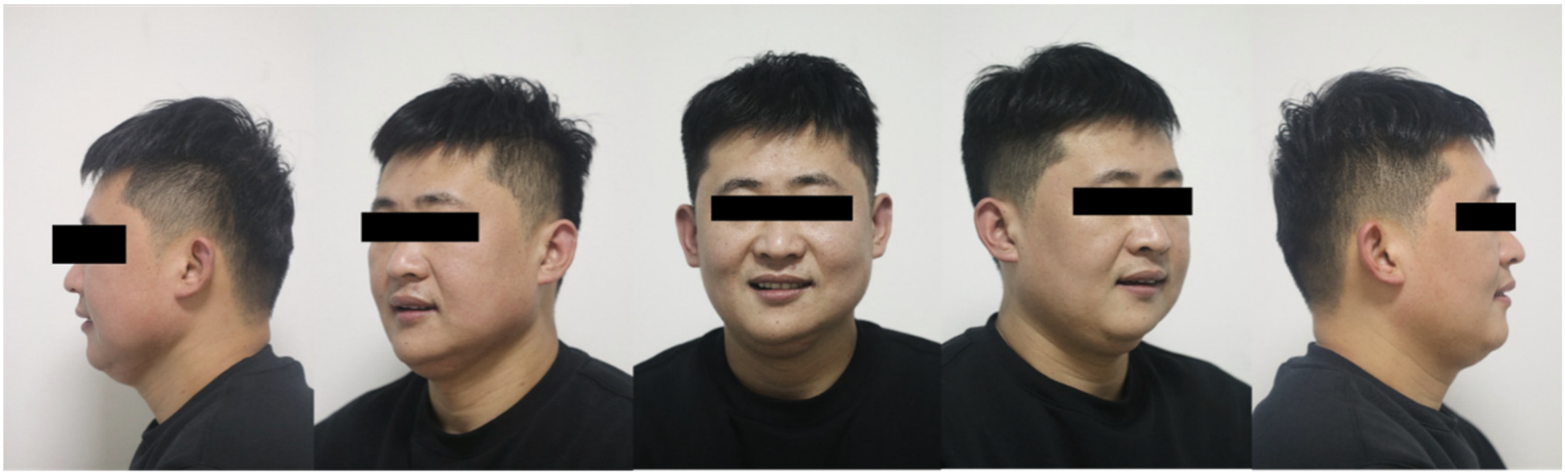
Facial view of the final restoration.

**Figure 11:**
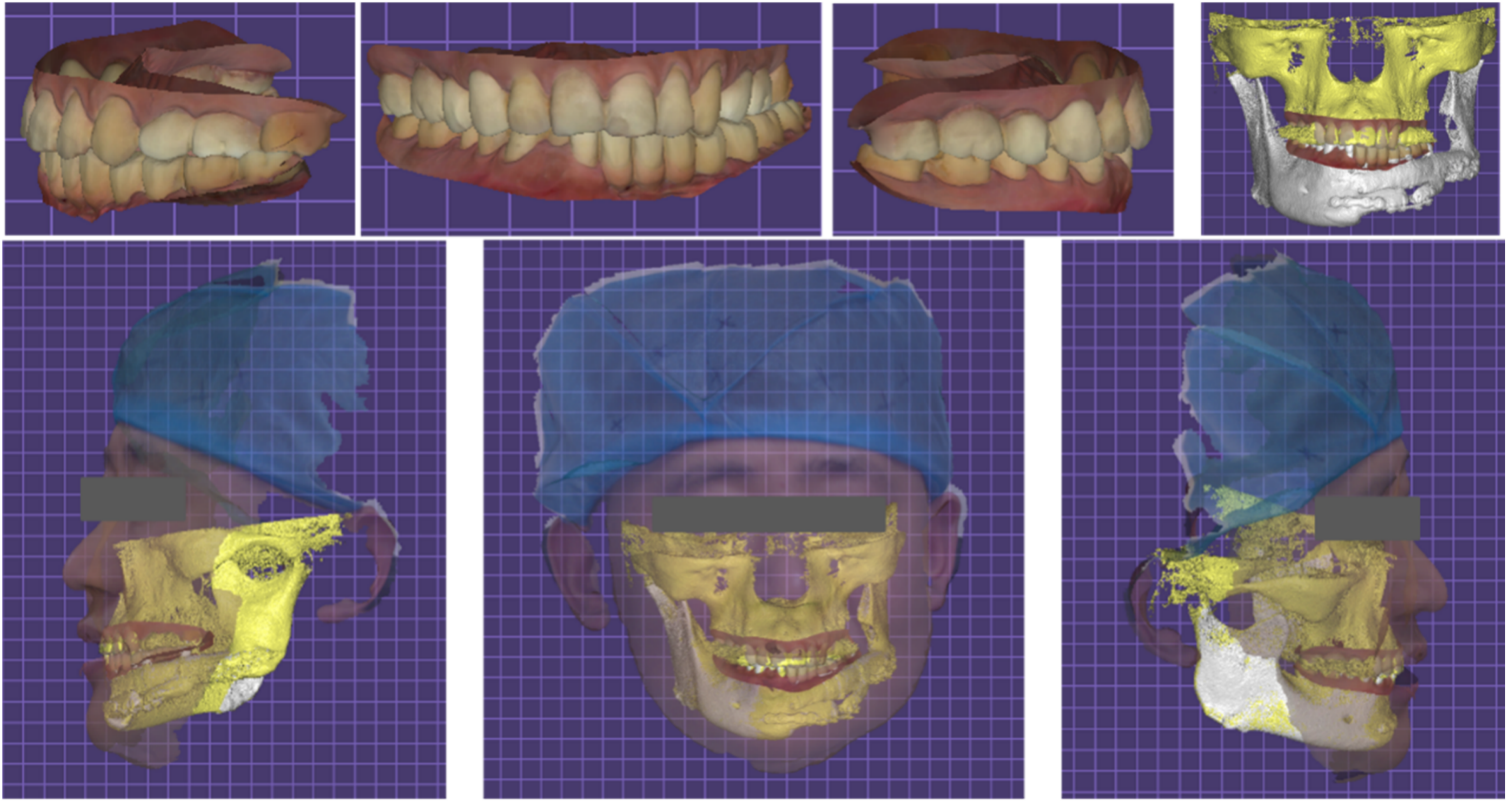
Facial scan after the completion of the final restoration and electronic facebow recording of mandibular movement trajectory.

After the final prosthesis was completed, a fitting trial was conducted. During the try-in of the final restoration, occlusal paper was utilized to assess the occlusal relationship. The patient was instructed to perform centric occlusion as well as protrusive and lateral movements, with adjustments made for any high points. The final restoration, a pure titanium implant bridge, was sent back to the lab for high polishing and factory bonding to mitigate issues with residual adhesive during clinical bonding. The final fitting was successfully completed (Figure 8D–I). Following the treatment procedure, the patient's facial appearance was documented through photography, and additional examinations were conducted, including an electronic facebow assessment of the patient's occlusal movements. Intraoral scanning technology was employed to evaluate the state and distribution of occlusal contacts across the entire dental arch. The patient reported a satisfactory outcome from the restoration, and the final restoration was completed (Figures 9–11). Follow-up 6 months later, the patient has no obvious discomfort, chewing, pronunciation, and aesthetics are satisfactory, occlusion is normal, there is no popping of joints, there is no abnormality in the trajectory of mandibular movement, and there is no obvious redness and swelling of the gingiva, and the patient is instructed to maintain oral hygiene, periodontal scaling on a regular basis, and regular review.

## Discussion

Primary treatment of enamel cell tumors is predominantly surgical, and the usual treatment for recurrence remains the surgical approach. In recent years there have been suggestions in the literature that carbon ion radiotherapy may be an effective treatment option for local recurrence of enamel cell tumors after surgery (13). Radiation therapy offers additional opportunities for patients with inadequate margins and those with metastatic disease (14, 15). In the present case, after communication with the patient, the extra-oral surgeon opted for extended resection of the mass and segmental resection of the mandible, with no recurrence at 1-year postoperative follow-up and no radiotherapy. Due to jawbone defects caused by tumors and other factors, patients often face challenges related to aesthetics, function, and mental health. Free fibula myocutaneous flap reconstruction of mandibular abnormalities has emerged as a popular and highly successful surgical procedure that restores the patient's functional skills and facial look. The restoration of fundamental abilities including speaking, chewing, and appearance is the main objective of oral rehabilitation. But once a tumor is removed, the reconstruction process usually includes several consecutive tooth loss and changes to the oral cavity's soft and hard tissue components, creating a highly specialized environment. Numerous answers to these problems are provided by the developments in implant technology (8). Achieving a durable and effective restorative outcome is closely linked to the technique used for impression taking, the assessment of the mandibular occlusal relationship and movement pathways, as well as soft tissue grafting.

This case was referred to our department via extraoral fibular implantation with buccal deviation of the grafted bone, high anterior and narrow posterior occlusal space, facial asymmetry, and tilted implant angle. Considering the lack of restorative space and the sufficient number of implants in this patient, the implant-retained restorative approach became the final choice. Implant-supported ceramic crowns provide high retention and low complication rates, along with excellent aesthetic results (16). Creating healthy soft tissue cuff structures and establishing a suitable occlusal connection with any remaining teeth are critical steps in the implant restoration procedure for patients undergoing bone reconstruction. To achieve long-term restorative success, these criteria allow patients to properly manage plaque control surrounding the crowns and adjacent soft tissues (17). The techniques employed in soft tissue grafting, the identification of mandibular occlusal connections and movement trajectories, and the process of taking impressions are closely associated with this procedure.

For taking impressions in edentulous jaws and with numerous implants, conventional methods like using a window tray and silicone materials remain the gold standard. However, these traditional techniques are less suitable for the distinct mucosal structures that arise after fibula grafting because they are technically delicate, difficult to execute, and prone to distortion during transit (18). Intraoral scanning technology, serving as the core of CAD/CAM technology, possesses a greater degree of comfort in comparison with traditional impression-taking techniques while capturing intraoral information (19, 20). Studies reveal that although the number of implants tends to increase the variance in digital scanning results, the differences are still statistically negligible. Moreover, there are clinically acceptable differences between the plaster models made from conventional imprints and the virtual models made from intraoral digital scans (21). In order to compare the data disparities between digital scanning methods and traditional techniques employing silicone impression materials followed by plaster models, Iwauchi et al. conducted a study involving eight patients. According to their findings, digital scanning techniques showed better accuracy (22). The ICam4D photogrammetry system provides a dependable method for assessing implant orientation, with an accuracy range of 2–203 μm (23). Photogrammetric technology can be used to assess implant position, evaluate implant restorations, measure peri-implant mucosal recession, verify the reliability of implant impressions, and quantify the three-dimensional surface morphology of implants (24, 25). Face scanners are particularly good at taking precise reference points, and when combined with CAD software, they may use these points to find virtual articulators with accuracy. As a result, it is possible to overlay data from intraoral digital scans onto facial scans and virtual face bows (26). By combining the soft tissue structures of the face, this integration makes it possible to create customized restoration designs that meet both practical and cosmetic requirements (27).

Mandibular movement recording is an essential tool for evaluating the functional effectiveness and overall health of the stomatognathic system (28), this is especially significant for the temporomandibular joint (TMJ) in patients undergoing bone grafting. The electronic face bow uses recorded mandibular movement trajectories to alter dynamic occlusion, effectively conveying jaw connections with simplicity, accuracy, and speed. Electronic face bows assist in producing restorations that can more precisely restore patients' occlusal functions while streamlining the therapeutic process, cutting down on clinical adjustment times, and minimizing medical expenses (29). The digital face bow can record the range and trajectory of mandibular motions in real-time throughout the functional reconstruction stage (30). Subsequently, the gathered information is combined with 3Shape and Exocad software to enable the digital creation and fabrication of the ultimate restoration. Restorations may now more closely match pre-existing occlusal relationships and intraoral structures thanks to the computerized workflow, which also greatly speeds up clinical adjustment times and improves treatment effectiveness. By offering both theoretical insights and data-driven references for the precise restoration of occlusal function in posterior fixed prostheses, this technique increases restoration precision. Using facial bow to record mandibular movement data while the patient tried on the temporary restoration, the treatment plan was customized in this instance. The restoration was then designed by integrating this data with digital data. In the end, the finished restoration guaranteed patient comfort and needed little time for correction. ICam4D photogrammetry, electronic face bows, oral scanning, facial electronic scanning, and professional design tools were all used in this process. Through the use of a fixed temporary restoration and this integration, a fully digital restorative treatment process that is managed was achieved. Furthermore, a three-dimensional virtual patient was generated by combining occlusal connections and facial morphology for a thorough assessment.

Following jawbone grafting, inflammatory reactions or mucosal hyperplasia frequently occur in the soft tissue lining of free flaps, resulting in long-term non-keratinized mucosa (31). For dental implants to remain stable over the long term, sufficient keratinized gingiva breadth is essential (32). When alveolar bone defects occur, inadequate keratinized mucosa presents a serious concern for implant-fixed restorations and bone reconstruction. Implants arising from thin or mobile mucosal tissues should undergo soft tissue grafting (33) because mucosal tissue from fibula grafts frequently fails to develop adequate keratinized mucosa and lacks attached gingiva, which can cause instability on the implant surface (34) and an increased risk of peri-implantitis (35). Current techniques include subepithelial connective tissue graft (SCTG), free gingival graft (FGG), apically repositioned flap (ARF), and soft tissue substitutes to address inadequate keratinized gingiva width and height around implants (36), Following fibula grafting, regions without keratinized gingiva are not appropriate for ARF or SCTG. Although FGG can produce consistent outcomes and a large increase in keratinized gingiva, it necessitates a second surgical site, which complicates the surgery and may exacerbate postoperative responses. Additionally, the availability of graft material is limited (37). On the other hand, a second surgical site is not necessary for the soft tissue augmentation process of ADM (acellular dermal matrix) grafting. After a year, follow-up research has revealed no statistically significant differences between ADM and SCTG in terms of soft tissue augmentation results (38). In this example, the patient came with alveolar bone abnormalities and many missing teeth, with the palate failing to generate sufficient keratinized mucosa. Therefore, decellularized dermal matrix grafting was performed, coupled with local fixation of mucosa and regeneration of keratinized tissue, aiming for a favorable prognosis.

There are still shortcomings in this case, such as bone reconstruction without a restorative-oriented approach. Surgical guides and implant guides were not used for preoperative planning due to a variety of factors, including patient preference and economic considerations. Although the use of digital impression technology and electronic face arches helps to simplify the process and improve patient comfort while maintaining impression accuracy, they do not solve the problem of suboptimal implant angulation. Since implant denture restorations should ideally ensure occlusal contact within the implant diameter, the final decision for a molar restoration is to use an angulation-free composite abutment and establish a unilateral posterior partnership. The accuracy and final outcome of bone reconstruction and implant restorations may be greatly improved if the patient and surgeon develop an awareness of digital restorations at the first visit. Recent studies have shown good prognostic and restorative outcomes with immediate implantation of fibular reconstruction after benign and malignant tumors, taking into account the effects of radiation, management of the soft skin tissues, and implantation time and stability of the fibula for the convenience of patients (5, 39). Kim et al. analysed several cases and found that patients with oral cancer who received immediate implants during jaw reconstruction had a lower risk of radiolucent osteonecrosis and implant failure (40). Chang et al. achieved good clinical results with the application of fibula cutting guides and implant position drilling guides, light-curing models, etc. (41). By allowing digital tooth arrangement to establish the final prosthesis shape, preoperative visualization of the bone reconstruction design can decrease surgery time, increase the accuracy of maxillary reconstruction, and serve as a reference for implant placement. One independent risk factor affecting the integration of the implant during oral restoration is its orientation. More accurate implant placement made possible by the use of a surgical guide can result in instant prosthesis restoration after implantation, cutting down on treatment time and perhaps preventing soft tissue overgrowth (42). Nevertheless, there are drawbacks to using digital technologies in the repair process. For instance, the electronic facebow still requires several visits to finish the restoration because it does not completely overcome the limitations of the classic facebow with regard to tooth position requirements. The problem of the high number of visits and the long treatment period still exists. Furthermore, the mucosal shape under pressure cannot be captured by digital three-dimensional scanning technology, which limits its use in implant-supported prostheses and limits the alternatives for repair. In the future, better restoration results are probably going to result from the combination of digital technology and a restoration-focused treatment strategy.

## Conclusion

This case creates a three-dimensional virtual patient through facial scanning, intraoral scanning, ICam4D external oral scanning, and electronic facial arch technology, restores the patient's bite function, and reduces the clinical operation time. It can be used as a feasible treatment plan for oral repair after mandibular fibula transplantation.

## Data Availability

The raw data supporting the conclusions of this article will be made available by the authors, without undue reservation.
